# Update for Anaesthetists on Clinical Features of COVID-19 Patients and Relevant Management

**DOI:** 10.3390/jcm9051495

**Published:** 2020-05-15

**Authors:** Benedikt Preckel, Marcus J. Schultz, Alexander P. Vlaar, Abraham H. Hulst, Jeroen Hermanides, Menno D. de Jong, Wolfgang S. Schlack, Markus F. Stevens, Robert P. Weenink, Markus W. Hollmann

**Affiliations:** 1 Department of Anesthesiology, Amsterdam University Medical Centers, Location AMC, Meibergdreef 9, 1105 AZ Amsterdam, The Netherlands; a.h.hulst@amsterdamumc.nl (A.H.H.); w.s.schlack@amsterdamumc.nl (W.S.S.); m.f.stevens@amsterdamumc.nl (M.F.S.); r.p.weenink@amsterdamumc.nl (R.P.W.); m.w.hollmann@amsterdamumc.nl (M.W.H.); 2 Laboratory of Experimental Intensive Care and Anesthesiology (L·E·I·C·A), Amsterdam University Medical Centers, Location AMC, 1105 AZ Amsterdam, The Netherlands; m.j.schultz@amsterdamumc.nl (M.J.S.); a.p.vlaar@amsterdamumc.nl (A.P.V.); 3 Department of Intensive Care, and Laboratory of Experimental Intensive Care and Anesthesiology (L·E·I·C·A), Amsterdam University Medical Centers, Location AMC, 1105 AZ Amsterdam, The Netherlands; 4 Mahidol–Oxford Tropical Medicine Research Unit (MORU), Faculty of Tropical Medicine, Mahidol University, 420/6 Rajvithi Road, Bangkok 10400, Thailand; 5 Nuffield Department of Medicine, University of Oxford, Old Road Campus Research Build, Roosevelt Dr, Headington, Oxford OX3 7DQ, UK; 6 Department of Medical Microbiology & Infection prevention, Amsterdam University Medical Centers, Location AMC, 1105 AZ Amsterdam, The Netherlands; m.d.dejong@amsterdamumc.nl

**Keywords:** COVID-19, SARS-CoV-2, ventilation, emergency care, intubation, antiviral therapy

## Abstract

When preparing for the outbreak of severe acute respiratory syndrome coronavirus 2 (SARS-CoV-2) infection and the coronavirus infection disease (COVID-19) questions arose regarding various aspects concerning the anaesthetist. When reviewing the literature it became obvious that keeping up-to-date with all relevant publications is almost impossible. We searched for and summarised clinically relevant topics that could help making clinical decisions. This is a subjective analysis of literature concerning specific topics raised in our daily practice (e.g., clinical features of COVID-19 patients; ventilation of the critically ill COVID-19 patient; diagnostic of infection with SARS-CoV-2; stability of the virus; Covid-19 in specific patient populations, e.g., paediatrics, immunosuppressed patients, patients with hypertension, diabetes mellitus, kidney or liver disease; co-medication with non-steroidal anti-inflammatory drugs (NSAIDs); antiviral treatment) and we believe that these answers help colleagues in clinical decision-making. With ongoing treatment of severely ill COVID-19 patients other questions will come up. While respective guidelines on these topics will serve clinicians in clinical practice, regularly updating all guidelines concerning COVID-19 will be a necessary, although challenging task in the upcoming weeks and months. All recommendations during the current extremely rapid development of knowledge must be evaluated on a daily basis, as suggestions made today may be out-dated with the new evidence available tomorrow.

## 1. Introduction

The severe acute respiratory syndrome coronavirus 2 (SARS-CoV-2) outbreak emerged in Wuhan, China on 31 December 2019 and the resulting coronavirus infection disease (COVID-19) has been declared a pandemic by the World Health Organization on 11 March 2020. To date, four different common coronaviruses are known causing for millions of cases of respiratory infections all over the world. The SARS-CoV-2 is a single-stranded RNA virus and has, based on decoration with glycoprotein spikes, a crown-like membrane. The nucleocapsid includes proteins and the RNA genome, which encodes polyproteins, e.g., replicase, proteases and structural proteins. All these proteins can interfere with the human innate immune system. Angiotensin converting enzyme 2 (ACE2), a cellular receptor present in the lung, binds to viral proteins and serves as entry for the virus after host-viral membrane fusion. Subsequently, after endocytosis, the RNA genome is released into the host cell and translated and replicated within the cell. Detection of virus RNA by real-time reverse transcriptase polymerase chain reaction (RT-PCR)-based methods is a keystone in diagnosing current infection with SAS-CoV-2, while detection of immunoglobulins helps to test for previous infections and immunity. The latter can in the future hopefully be achieved by adequate vaccination, and different strategies are currently explored to find a respective vaccine, e.g., live attenuated vaccines, viral vector-based vaccines, recombinant protein-based vaccines, DNA or messenger RNA vaccines.

Yearly, up to 2.6 million people die from respiratory infections, and the consequences of SARS-CoV-2 infection should be interpreted in the light of these numbers [1,2]. Programmatic recommendations have been published on how to optimize perioperative infection control and operating room management [3]. When preparing for the outbreak in the Netherlands, including training of staff (physicians and nurses) for tasks that are normally outside their working profile and comfort zone, questions arose regarding various aspects of SARS-CoV-2 infection and the coronavirus infection disease (COVID-19). When reviewing the literature it became obvious that keeping up-to-date with all relevant publications is almost impossible: within the first ten weeks of 2020, more than 1000 articles related to COVID-19 have been listed in PubMed, and during preparation of this manuscript over the last 12 days another 1000 publications have been published. Not all of these publications can possibly have gone through a thorough review process, and indeed falsified publications were presented and quickly retracted, including publications in journals with high impact factors [2]. We are aware that we are also prone to include misinformation and selection bias with respect to our topics chosen, thus, we remind the reader to be critical of the information provided in our article as well. Nevertheless, we searched for and summarised clinically relevant topics from publications listed in PubMed before 31 March 2020 that could help making clinical decisions. This is a subjective analysis of literature concerning specific topics raised in our daily practice, and we did not intend to perform a systematic review on all topics concerning COVID-19.

## 2. Clinical Features of Patients Presenting with COVID-19 in the Emergency Department and Indication for Endotracheal Intubation

Clinically severe infections (in aged but also in younger patients) with a wide diversity of presenting symptoms, including altered mental status, myocardial, hepatic and kidney injury, and gastrointestinal symptoms like prolonged periods of nausea, vomiting and diarrhoea have been described. Most patients infected with SARS-CoV-2 have mild respiratory symptoms and a good prognosis after infection; these patients will not be presented to the anaesthetist or intensive care physician. However, some patients develop severe hypoxic respiratory failure early in the disease course, making non-invasive or invasive application of oxygen necessary. Next to hypoxemia, exhaustion from respiratory efforts may also be an indication for early endotracheal intubation.

Any application of oxygen carries a significant risk of aerosolisation of virus material and, thereby, potentially disease transmission [4]. Preliminary observations from Italy suggest a poor response of COVID-19 patients to non-invasive ventilation [4]. Invasive ventilation is associated with reduced aerosolisation. Delaying unavoidable tracheal intubation should be prevented, while delaying avoidable tracheal intubation may be beneficial [5,6]. Adequate precautions to prevent virus transmission during intubation of a SARS-CoV-2 positive or suspected patient have been described recently and should be adapted to local circumstances [7]. Cough is one of the major methods of human-to-human viral spread. Any airway instrumentation, insertion of gastric tubes and drugs used during induction of anaesthesia (e.g., opioids) can exacerbate coughing and, thereby, viral transmission. Coughing and bucking are prevalent events during intubation and emergence from anaesthesia. The use of intravenous lidocaine perioperatively is an effective, simple and safe means to decrease coughing without increased risk of harm [8]. Dexmedetomidine, remifentanil or fentanyl are other options to reduce coughing, which are at least equally effective, but with haemodynamic and respiratory side effects [9].

## 3. Ventilation in the Critically Ill COVID-19 Patient

Lungs of COVID-19 patients are characterised by the coexistence of severely affected lung areas with atelectasis that are not, or hardly, recruitable and adjacent to areas that are unaffected and remain remarkably compliant, which are at risk of over-distension. One important aim of invasive ventilation in general, including patients with COVID-19, is to prevent damage caused by the ventilator by protecting undamaged and otherwise fragile lung tissue. This overrules the aims of achieving normoxaemia and normocapnia.

Limiting tidal volumes (V_T_) to 6 mL/kg ideal body weight (IBW) can reduce mortality by up to 25% in patients with acute respiratory distress syndrome (ARDS) [10]. International guidelines, therefore, strongly recommend using limited V_T_, in particular for patients on assist ventilation (i.e., not on a pressure support mode). The use of a low V_T_ of 6 mL/kg IBW, or even lower, is probably also beneficial in COVID-19 patients. Of note, IBW is not the same as actual body weight, and is calculated from the height of a patient; a simple calculation for a woman is “height (in cm)—110” and for a man “height (in cm)—105”. Shortly after intubation and start of invasive ventilation, a default V_T_ of 350 mL in women and 400 in men turned out to be practical; this can be further adjusted after arrival in the ICU. When using a low V_T_, a higher respiratory rate, sometimes up to 35–40 per minute, has to be used to achieve an adequate minute volume. This high respiratory rate can generally be accepted, even when an increase in “dead space ventilation” results in hypercapnia, as long as the blood pH remains above 7.2 (i.e., “permissive hypercapnia”). Use of a low V_T_ can also result in a slight deterioration in oxygenation, which can be compensated for with an increase in the inspired fraction of oxygen (FiO_2_). When the ventilator is converted from assist ventilation to pressure support ventilation, the V_T_ will increase, sometimes well above 6 mL/kg IBW, which can be accepted if the driving pressure (i.e., the level of pressure support) remains low.

Different morphologic phenotypes in ARDS exist: ‘focal, non-recruitable’ and ‘non-focal, recruitable’, needing different therapeutic approaches (see Table 1). COVID-19 pneumonia and ARDS most likely presents like a ‘focal, non-recruitable’ ARDS in most cases. However, in some patients COVID-19 also might present as ‘non-focal, recruitable lesions’ ARDS, as well at the start of the illness as later on during the process.

Patients with ARDS need more positive end-expiratory pressure (PEEP) than patients without ARDS, usually with an initial setting of 10 cm H_2_O. Use of PEEP recruits collapsed lung areas and also keeps these areas partially open. However, aggressive lung recruitment manoeuvres with use of ‘preventive high PEEP’, with an intention to maximally recruit lung parenchyma, is harmful and associated with excess mortality in patients with ARDS [11]. A major drawback of PEEP is overdistension in more compliant parts of the lung. As stated above, this seems an even more important issue in COVID-19 patients than in patients with non-COVID-19 ARDS.

One recent study that compared ventilation with PEEP according to a low PEEP and FiO_2_ table, with a personalized approach based on lung morphology in which patients with ‘focal’ ARDS received low PEEP in the prone position, and patients with ‘non-focal’ ARDS received recruitment manoeuvres and high PEEP [12], suggesting a benefit of ventilation with low PEEP and the prone position in patients with ‘focal’ ARDS. While COVID-19 lesions appear rather ‘non-focal’, they behave like ‘focal’ lesions as they seem non-recruitable. Therefore, a ventilation strategy with PEEP according to a low PEEP and FiO_2_ table in combination with early prone positioning might be preferred. PEEP can lead to better oxygenation, which can be falsely interpreted as a ‘success’ of the intervention, because it signifies successful lung recruitment. However, it is important to remember that oxygenation is not the only goal, and that lungs of patients with COVID-19 are characterised by non- or hardly-recruitable, severely affected lung areas. Recruitment through increasing PEEP should only be pursued in case of severe hypoxaemia that does not respond to an increase in FiO_2_ well above 0.6. Too high PEEP (>10 to 12 H_2_O) will cause overdistension of unaffected lung areas, which can then result in an increase in driving pressure. For this reason the recommendation is to apply not more than 10–12 cm H_2_O PEEP, and only use higher PEEP levels when concomitant driving pressure is reduced. Last but not least, PEEP levels > 10 cm H_2_O can compromise cardiac performance, with negative effects on preload, resulting in reduced blood pressure and a possibly increased need for vasopressors and intravenous volume, which are both associated with increased morbidity in ARDS patients. For COVID-19 patients requiring large doses of vasopressors to maintain an adequate blood pressure, excessive PEEP should be reconsidered.

COVID-19 patients show next to the affected lung areas, remarkably compliant unaffected lung areas, which can be ventilated with driving pressure well below 15 cm H_2_O, often as low as 5–7 cm H_2_O. The driving pressure, which is by approximation the difference between the maximum airway pressure (in pressure controlled ventilation) or the plateau pressure (in volume controlled ventilation), and the PEEP level, is associated with patient outcome [13]. Driving pressure can be regarded as an overall marker of lung damage, but can also be iatrogenic resulting from too high a V_T_ or excessive PEEP. While there are currently no randomised studies, neither in patients with ARDS due to other diseases nor in COVID-19 patients, on interventions targeting driving pressure, we favour ventilation with a low driving pressure where possible. The easiest way to lower the driving pressure is to (further) limit V_T_. A reduction in PEEP may also have a beneficial effect on the driving pressure.

Prone positioning improves outcomes of patients with ARDS [14]. It results in significant improvement in oxygenation, probably due to a reduction in shunt by improving ventilation-perfusion matching, but more importantly leads to a more homogenous distribution of inspired air with each breath provided by the ventilator. Sessions of prone positioning should be sufficiently long, if possible at least 16 h or more per day. This means that the patient is kept only briefly (i.e., for a few hours) in a supine position. Within this regimen the timing of turning the patient in the prone position and back can be flexible. Existing literature suggests no differences in mortality between so-called ‘responders’ (patients who show an improvement in oxygenation in the prone position) and ‘non-responders’ (those who do not show an improvement) [15,16]. Once prone positioning has started, the decision on continuation of this intervention should not be based on the response in a single session. Prone ventilation does not necessarily require additional sedation, and additional neuromuscular blockade in sedated patients is generally not needed. However, COVID-19 patients can respond to ‘turning’ (from supine to prone, and vice versa), with coughing and severe asynchrony, resulting in, at times, deep desaturations, that make administration of additional sedatives, opioids, and eventually neuromuscular blockade necessary. Short time use of pressure support ventilation with the patient triggering the machine could be an alternative strategy to tackle ‘turning’ induced sequelae.

We prefer to closely monitor the above, preferably with each nursing shift and maybe even more frequently. A practical list of what to monitor, and how to improve settings is provided in Table 2.

## 4. What are the Roles of PCR, Antibody Detection and Chest Computer Tomography (CT) in Making the Diagnosis SARS-CoV-2 Infection?

**Figure 1:**
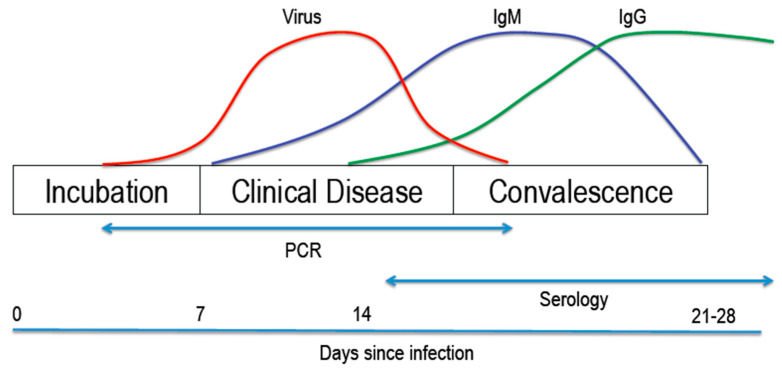
Detection of the virus in clinical specimens or detection of specific antibodies in blood. PCR: polymerase chain reaction, IgM: Immunoglobulin M; IgG: Immunoglobulin G.

As for other respiratory viral infections, laboratory evidence of SARS-CoV-2 infection can be provided directly, by detection of the virus in clinical specimens, or indirectly, by detection of specific antibodies in blood. The benefits of both diagnostic strategies depend on the stage of the infection (Figure 1): viral shedding begins to occur shortly before onset of symptoms during the incubation period, increasing to peak levels during the acute illness, while it takes several days to weeks until virus-specific antibodies can be detected.

For this reason, the current recommended strategy for laboratory diagnosis of COVID-19 during the acute stage of the infection is the detection of SARS-CoV-2 RNA in clinical specimens by real-time reverse transcriptase polymerase chain reaction (RT-PCR)-based methods. Based on viral genetic sequences from the initial isolated viruses during the early stages of the epidemic, highly sensitive and specific assays were immediately developed, targeted at genes encoding for the viral RNA-dependent RNA polymerase (RdRp) and envelope [17]. This assay has been adopted by the World Health Organisation and formed the basis of diagnostic testing in many countries worldwide. In addition, the United States Center of Disease Control (CDC) has developed a nucleocapsid-targeted assay for use by public health and other qualified laboratories in the United States [18], whilst a rapidly increasing number of commercial molecular assays are in development or entering the market [19]. In addition to PCR-based virus detection assays, rapid tests based on detection of viral antigens are in development. Such assays have the advantage of providing a result within minutes rather than hours but, similar to rapid antigen tests for other respiratory viruses, limited sensitivity likely will be an issue [20,21].

The sensitivity of PCR- or antigen-based virus detection assays is dependent on the quality of the specimen, hence adequate instruction and training in collection of specimens is essential. Nasopharyngeal or combined nasal- and oro-pharyngeal swabs are the preferred specimens for SARS-CoV-2 testing. Ideally, flocked swabs should be used as these increases the yield of cellular material, whilst swabs containing cotton or wood should be avoided as these may inhibit PCR reagents. After collection, swabs for PCR testing should be transferred to a transport medium that preserves viral RNA.

Sensitivity of PCR-based diagnostics of upper respiratory tract specimens seems generally high during the acute stage of infection. Compared to mild infections, severe COVID-19 is associated with prolonged duration (longer than 10 days from illness onset) and higher levels of viral RNA [22]. However, false-negative test results are observed, especially during the later stages of the illness when viral loads are dropping, including in patients with progressive disease. In these circumstances, collection of lower respiratory tract specimens, such as sputum, endotracheal aspirates or broncho-alveolar lavages should strongly be considered, as detection rates in these specimens may be higher [23]. In addition to these specimens, viral RNA may also be detected for prolonged periods in faecal or rectal specimens [24]. Virus has not been detected in urine samples and rarely in blood specimens.

Serological assays that detect virus-specific antibodies, such as immunoglobulin (Ig) A (IgA), IgM or IgG, in blood or saliva are essential to diagnose recent or past infections; their diagnostic role during the acute stage of infection, early after symptom onset, is limited. Potential pitfalls of antibody detection assays include limited specificity, for example due to cross-reactivity with antibodies to other human coronaviruses, as well as limited sensitivity, which may pose a particular concern in diagnosing mild infections which may be associated with weaker antibody responses [25]. Although detected antibodies likely protect from re-infection, investigations are needed and ongoing to correlate IgG titres to virus neutralizing capacity, indicative of protective immunity. In addition, research is needed to assess the longevity of protective immunity. Many commercial serological assays are currently in development, several of which are entering the market [19,25,26].

Studies evaluating the diagnostic value of IgM and IgG detection in COVID-19 patients showed higher sensitivity of PCR during the first 5–7 days after illness onset and higher sensitivity of IgM detection thereafter [27,28]. Highest sensitivities were observed when combining PCR and IgM detection. Sars-C0V-2-specific IgG antibodies were first detected after a median period of 14 days after illness onset in both studies [27,28].

In summary, while PCR detection of viral RNA in respiratory specimens remains the mainstay of diagnostics in patients presenting with suspected COVID-19, there may be a role for serological tests in patients presenting late during the course of infection, for example with progressive or complicated disease, at a time when viral loads may have dropped, especially in the nasopharynx, while an antibody response is evolving (Figure 1). In addition, antibody assays are of course essential for epidemiologic studies, surveillance of population immunity, vaccine response studies and, for example, risk assessments for health care workers.

There is an ongoing discussion as to whether chest computer tomography (CT) can reliably prove SARS-CoV-2 infection and COVID-19, and whether every patient undergoing surgery needs to have a chest CT before surgery. Up to now, the role of CT scan for diagnosing COVID-19 is founded on a very limited number of patients and might be useful in patients with a clinically-suspected SARS-CoV-2 infection and a negative RT-PCR test [29]. Different scenarios have been described: chest CT may be negative for viral pneumonia in clinically symptomatic patients at initial presentation [30,31], but on the other hand patients who had negative RT-PCR for COVID-19 at initial presentation showed chest CT findings typical of viral pneumonia [32]. A possible higher prevalence of influenza than COVID-19 additionally limits the specificity of CT for diagnosis of COVID-19. Chest CTs from COVID-19 positive patients and non-COVID viral pneumonia patients were analysed by Chinese and United States radiologists, showing sensitivities of 72–94% and specificities of 24–100% in differentiating COVID-19 from non-COVID-19 pneumonia. The most discriminating features for COVID-19 pneumonia included a peripheral distribution, ground-glass opacity and vascular thickening [33]. A fully automatic framework and accurate detection of COVID-19 using chest CT has been developed using a deep learning model COVID-19 detection neural network (COVNet) [34]. Based on 4356 chest CT exams from 3322 patients, COVNet’s per-exam sensitivity and specificity for detecting COVID-19 in an independent test set was 114 of 127 (90% (95% CI: 83%, 94%)) and 294 of 307 (96% (95% CI: 93%, 98%)), respectively. The per-exam sensitivity and specificity for detecting community-acquired pneumonia (CAP) in the independent test set was 87% (152 of 175) and 92% (239 of 259), respectively [34]. The sensitivity of chest CT in patients with clinical and epidemiologic features compatible with COVID-19 infection was reported to be higher (98%) than that of RT-PCR (71%) [35]. However, these data were obtained from only 51 patients and cannot be extrapolated to clinically asymptomatic patients.

In light of these data the CDC does not currently recommend chest CT to diagnose COVID-19. Viral testing remains the only *specific* method of diagnosis [36]. Confirmation with the viral test is required, even if radiologic findings are suggestive of COVID-19 on CXR or CT. The American College of Radiologists states that the findings on chest imaging in COVID-19 are generally not specific, and overlap exists with other infections, including influenza, H1N1, SARS and MERS. The UK Royal College of Radiologists stated on March 27th that “the use of additional chest CT to assess for the presence of likely COVID-19 infection may have a role in stratifying risk in patients presenting acutely and requiring a CT abdomen, particularly those needing emergency surgery. In the absence of rapid access to other forms of COVID testing, this is appropriate if it will change the management of the patient. However, a negative scan would not exclude COVID-19 infection” [37].

Anosmia is increasingly recognised as a symptom in COVID-19 infection. It can accompany other mild respiratory symptoms, or can present as an isolated finding [38]. In a European study, 80% of hospitalised patients of laboratory confirmed COVID-19 had anosmia at some point in the course of the disease [39]. It has been suggested that patients with isolated new-onset anosmia should be treated as suspected for COVID-19 [40].

## 5. How Long is the Virus Stable in Aerosol and on Surfaces?

The SARS-CoV-2 has an extreme transmissibility, and even asymptomatic people can transmit the infection [41]. High viral loads were detected soon after symptom onset, with higher viral loads detected in the nose than in the throat; viral load in the asymptomatic patient was similar to that in symptomatic patients [42]. Transmission occurred mainly after a couple of days of illness and was associated with modest viral loads in the respiratory tract, with viral loads peaking approximately 10 days after symptom onset [42]. Significant environmental contamination has been demonstrated not only through respiratory droplets but also by faecal shedding from patients with SARS-CoV-2 infection [43]. Thus, strict adherence to hand hygiene and decontamination of environment and equipment by routine cleaning is mandatory. This is of special interest after aerosol-forming treatments, e.g., endotracheal intubation. Different protection strategies for staff during endotracheal intubation have been described, and management of anaesthesia induction including protection strategies to prevent contamination of the OR environment are keystones to prevent medical staff infection [3,7]. SARS-CoV-2 has been shown to remain viable in aerosols at least a couple of hours, with a small reduction in infectious titre during the first 3 h [44]. The virus was more stable on plastic and stainless steel than on copper and cardboard; most relevant: viable virus was detected (in a greatly reduced virus titre) up to 72 h after application to these surfaces. However, this study did not investigate transmissibility from these surfaces to humans.

## 6. Paediatric Considerations: How are Children Involved in SARS-CoV-2 Infection?

A much higher prevalence of influenza than COVID-19 during the winter period made pneumonia as a result of other than SARS-CoV-2 infection likely during the beginning of the pandemic. This holds in particular true for children, infants and neonates: neonatal respiratory failure can result from a wide range of causes, and infection with other viruses are likely in this patient population [45].

In the beginning of the pandemic it seemed that children were spared from COVID-19, but recent data show that children of all ages can be infected: a review of 45 publications revealed that 1–5% of the diagnosed COVID-19 cases were children, with more asymptomatic cases than in adults [46]. Recent data from the CDC found 1.7% of 149,082 cases diagnosed in the USA were children, while about 20% of the population are children. Of those diagnosed 27% did not have any of three cardinal symptoms (fever, cough, shortness of breath) while this percentage in the adult population was 7% [47]. While most children had fairly mild symptoms and a hospital admission percentage of 10% in infants under one year the hospitalisation rate was above 50%. The percentage range of children admitted to the PICU was calculated to be 0.6–2.0%. In a Chinese cohort of 33 neonates with or at risk of COVID-19, clinical symptoms were mild and outcomes were favourable [48]. Reasons for the mild presentation of COVID-19 in most children remains still unclear, but might be based on differences in immunity and anatomy, co-exposure to other respiratory tract viruses limiting SARS-CoV-2 load or differences in ACE2 expression [49]. Fever and respiratory tract symptoms are prevalent, and only very few children develop severe pneumonia and ARDS. The incidence of severe and critical disease was 5.2% and 0.6% in the Wuhan population, respectively; death has been extremely rare [50]. Again, the age group with the highest incidence of severe or critical disease (10.6%) were infants under one year of age. Elevated inflammatory markers or lymphopenia were less frequent in children than in adults. However, cytokine storm was found in some patients, which appeared more serious in critically ill children [51]. All age groups can acquire COVID-19 infection including new-borns, while vertical intrauterine transmission is not commonly reported [46,50,52]. Three neonates presented with early-onset SARS-CoV-2 infection and symptomatic COVID-19, with the most seriously ill neonate probably being symptomatic from prematurity, asphyxia and sepsis. Tissue samples including amniotic fluid, cord blood and breast milk were (up until now) negative for SARS-CoV-2, but the vertical maternal–foetal transmission cannot be finally ruled out based on these data [53]. In a small cohort of ten neonates born out of mothers with acute SARS-CoV-2 infection around delivery, six were born prematurely, six had shortness of breath and seven had chest radiographic abnormalities. One neonate died and four had extended hospitalisation. All of them were tested negative for SARS-CoV-2 PCR [54].

Although disease transmission seem to be primarily by aerosol or fomite, recent reports indicated a high percentage of SARS-CoV-2 positive rectal swabs outlasting the nasopharyngeal swab up to 24 days (range 6–24) [55,56,57]. Thus, faecal-oral transmission seems possible. Worth emphasising, that the disease could start with abdominal pain, vomiting and headache in children.

Special attention should be paid to the emotional burden in caring for paediatric patients. The health professional might be more willing to accept non-adherence to hygiene rules in order to act faster in an emergency situation with a child, or to make life more comfortable for the child. Similarly, parental fears are keeping children out of hospitals and emergency rooms and leads to delays in not-COVID related therapies/operations urgently needed [58,59].

Although generally in (suspected) COVID-19 patients as few persons as possible should be attending during induction of anaesthesia, attending parents can help to increase comfort and cooperation of the child. Furthermore, parents who choose to attend an emergency procedure of their child reported that they felt it was beneficial for themselves and for the child [60].

The large proportion of asymptomatic children makes it difficult to identify paediatric patients, who might be a source for further transmission and spreading of the virus [61,62]. Children might have a high viral load [63] and may not understand the concept of social distancing, leading to significant risk of in hospital spreading of COVID-19 [64]. Thus, careful evaluation of the child’s medical history including possible exposure to infected persons, a restricted contact to health care workers and other patients, and a low-threshold for PCR testing in children are even more important than in adults.

## 7. Are Immunosuppression, Hypertension or Diabetes Mellitus Risk Factors for Severe Disease?

### 7.1. Immunosuppression

Preliminary data from Italy suggest that among patients who are followed-up for cirrhosis, transplantation, autoimmune liver disease, chemotherapy for hepatoblastoma, none (children and adults) developed a clinical pulmonary disease, despite some patients tested positive for SARS-CoV-2 [65]. Additionally, other coronaviruses have not shown to cause a more severe disease in immunosuppressed patients. For this family of viruses the host innate immune response appears to be the main driver of lung tissue damage during infection.

### 7.2. Hypertension

The SARS-CoV-2 gains entry to pulmonary cells after binding to membrane angiotensine converting enzyme (ACE) 2, which is a homologue of ACE1, the enzyme that converts angiotensin I to angiotensin II [66]. The role of ACE2 is to enzymatically cleave angiotensin II to angiotensin, but its biology and therapeutic potential is much less clear. Angiotensin receptor-blocker (ARB) drugs can substantially increase ACE2 expression. Enhanced ACE2 expression could theoretically increase human SARS-CoV-2 infectivity and illness severity. Interestingly, although hypertension is thought to increase the severity of COVID-19 illness, ACE2 expression typically is reduced in hypertension models. Nonetheless, current evidence suggests that increased ACE2 expression would not be beneficial in SARS-CoV-2 infection and could probably be harmful, although other authors suggest a protective role of up-regulated ACE2 expression [67]. Although increased ACE2 expression with ARBs has been demonstrated in the kidneys and the heart, it is not yet known whether it is also up-regulated in the lungs [68]. About 30% of patients hospitalised because of the virus have been shown to have hypertension, but it should be recognised that the prevalence of hypertension in adults of a respective age-matched population is around that same percentage. Thus, the hypothesis, that hypertension (and treatment with ACE inhibitors or ARB) increases the risk of COVID-19 infection and severity of illness is not justified from analysis of current data. It has been suggested that in SARS-CoV-2-infected patients, dosing of ARB drugs should not be escalated, nor should new ARB therapy be initiated. However, ARBs should not be discontinued prior to confirmation of the hypothesis that ACE2 overexpression is indeed harmful to COVID-19 illness [69]. Changes in antihypertensive treatment carries the risk of destabilizing blood pressure control. Withdrawal of anti-hypertensive agents might lead to severe morbidity (e.g., myocardial infarction, stroke) and is not recommended at this moment in COVID-19 patients [70].

### 7.3. Diabetes Mellitus

Published data (Table 3) suggests that the prevalence of diabetes mellitus (DM) is higher in patients with COVID-19 compared to the general population [71,72,73,74,75,76,77,78,79].

In China, the origin of the majority of available data today, researchers reported a prevalence of DM as high as 20% in COVID-19 patients [72,74], while the prevalence in the general population is estimated at 6.6% [80]. From these data, it could be inferred that patients with diabetes are at a greatly increased risk of contracting SARS-CoV-2 and developing COVID-19. Possibly, increased ACE2 expression in patients with DM contributes to this increased susceptibility [81].

**Figure 2:**
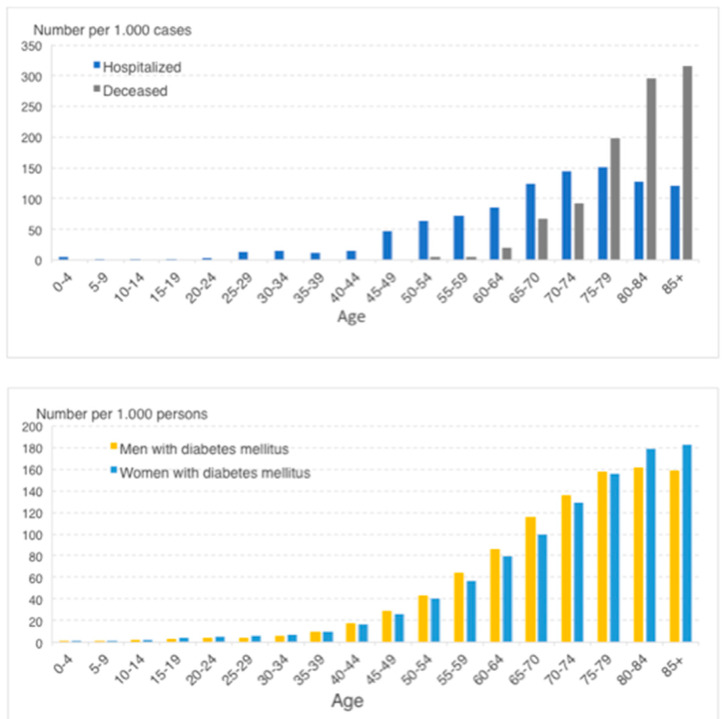
Upper panel: number of patients with DM (hospitalized and deceased) in patients with COVID-19 for different age groups; Lower panel: number of patients with DM in the general Dutch population for different age groups.

Even more, several studies show an increased risk or poor outcome for patients with DM [77,78,84]. Pathophysiologically, this is plausible because of the known increased risk of infection in patients with DM [85]. However, prevalence of DM also increases with advancing age and coincides with cardiovascular disease, all published potential risk factors for COVID-19 (Figure 2) [80]. It, therefore, remains to be established whether patients with DM are actually more susceptible to COVID-19 because of their diabetes. Nonetheless, in patients with severe acute respiratory syndrome (SARS) caused by the coronavirus SARS-CoV-2, diabetes and hyperglycaemia were identified as independent predictors for morbidity and mortality [85]. In addition, ACE2 expression is relatively high in the pancreas islets cells, possibly contributing to hyperglycaemia in COVID-19 patients and increased insulin need [86]. Generally spoken, for all hospitalised and ICU patients, DM is an independent risk factor of poor outcome [87]. Evidence that this relationship in COVID-19 patients would be different from other forms of critical illness is currently lacking. Therefore, adherence to current guidelines on glycaemic control strategies seems most prudent and logical (including frequent monitoring and administering intravenous insulin with a glucose target range of at least <180 mg/dL) [88].

## 8. Are Routine Antiviral Treatments Useful?

No verified antivirals specific to COVID-19 exist at present. The efficacy and safety of candidate drugs in the treatment of COVID-19 need to be confirmed in future preclinical and clinical trials. The transition from first respiratory symptoms to ARDS is probably based on uncontrolled cytokine release. Chloroquine has a promising inhibitory effect on cytokine release, however, its clinical use is limited and must be balanced against severe side effects: to be effective, chloroquine must be dosed in its toxic range. Hydroxychloroquine, which exhibits an antiviral effect highly similar to that of chloroquine, could attenuate the severe progression of COVID-19, inhibiting the cytokine storm by suppressing T cell activation. Hydroxychloroquine has a modulating effect on activated immune cells, downregulates the expression of Toll-like receptors and Toll-like receptor mediated signal transduction, and decreases the production of interleukin-6. However, clinical trials are necessary to confirm the hypothesis that hydroxychloroquine exerts beneficial effects in COVID-19 patients [89].

Identification of candidate drugs and potential drug combinations targeting SARS-CoV-2 employing network proximity analyses of drug targets and human CoV (HCoV)-host interactions in the human interactome revealed 16 potential anti-HCoV repurposable drugs [90]. Some of them were validated in human cell lines, including the angiotensin receptor blocker irbesartan, selective estrogen receptor modulators like equilin and toremifene, immunosuppressant or antineoplastic agents like sirolimus and mercaptopurine and anti-inflammatory drugs (e.g., melatonin and eplerenone), mesalazine, paroxetine and dactinomycin. Furthermore, quinacrine, carvedilol, colchicine, camphor, oxymetholone and emodin next to the combinations of sirolimus plus dactinomycin, toremifene plus emodin, and mercaptopurine plus melatonin were investigated. The antiviral substances ledipasvir or velpatasvir might combat the new coronavirus with minimal side effects (fatigue and headache), whereas the combinations velpatasvir/sofosbuvir and ledipasvir/sofosbuvir might be effective due to their dual inhibitory actions on two viral enzymes [91]. Remdesivir, an anti-Ebola nucleotide analogue was reported to be effective in preventing MERS-CoV replication, reducing severity of disease, viral replication and lung damage [92]. Combining anti-viral and inflammatory treatments might become another possibility to reduce virus load [93]. Although some case series on use of antiviral therapy have been promising [94], so far no sound evidence is available to use any of these drugs on a routine basis in severely ill COVID-19 patients. Remdesivir targets the viral RNA-dependent RNA polymerase, thereby inhibiting replication of SARS-CoV-2 [95]. Pre-publication of a double-blinded randomised clinical trial including 1063 seriously ill COVID-19 patients from the US, Asia and Europe revealed that remdesivir significantly shortened the course of disease by four days (from 15 days in the control group to 11 days in the remdesivir group). Also, mortality was reduced from 11.6 to 8 days. However, this result did not reach statistical significance. In contrast, results of a double-blinded randomised clinical trial including 237 severely COVID-19 infected patients from Hubei, China did not show a significant effect on clinical time course: remdesivir did not significantly hasten the time to clinical improvement [96]. Adverse events were not different between the remdesivir and placebo group, although treatment had to be stopped early because of adverse events in 12% of patients in the remdesivir group, compared to 5% of patients in the placebo group. Data of this study were criticised about the late application of remdesivir: it was probably started too late in the course of the disease to be effective. To summarise, although data on the effectivity of remdesivir in COVID-19 patients are controversial, its early use in severe COVID-19 patients might be promising and given the, based on current data, acceptable risk profile warrants further trials to investigate possible beneficial effects (numerous clinical trials are underway).

## 9. Are Kidney and Liver Function Compromised in the Course of Disease?

Renal involvement in COVID-19 patients may be caused by direct infection, or may have a multitude of other etiologies, as occurs in critical illness in general. The human kidney might be a specific target for SARS-CoV-2 infection: post-mortem, SARS-CoV-2 antigens accumulated in kidney tubules, suggesting that SARS-CoV-2 infects the human kidney directly, inducing acute kidney injury (AKI) and contributing to viral spreading in the body [67]. Early renal involvement in hospitalised COVID-19 patients has been reported: proteinuria and haematuria were found in patients on hospital admission and CT scan of the kidneys showed reduced density, suggestive of inflammation and oedema [67]. In 710 hospitalised patients from China, 44% of patients had proteinuria and 27% had hematuria on admission. Patients with kidney impairment on admission had significantly higher in-hospital death rates [97]. Case series from China suggest that frank AKI occurs in up to 7% of hospitalised patients [72], and up to 29% of critically ill patients [76]. In this latter study, 17% of critically ill patients required renal replacement therapy. Renal impairment may require dosing adjustment of drugs used in the treatment of COVID-19 patients, e.g., if chloroquine and hydrochloroquine are used, dosages should be reduced in patients with severely impaired renal function. Dosing of lopinavir/ritonavir does not depend on renal function. The impact of COVID-19 on patients with pre-existing renal disease is currently unclear. Very early data suggest that haemodialysis may not lead to an aggravated clinical course, and theoretically may lead to milder disease. This is hypothesised to be due to reduced function of the immune system, or washout of inflammatory mediators, leading to a decreased cytokine storm [67]. The effect of pharmacological immunosuppression, for instance in transplant recipients, is currently unknown [67], one report from Italy suggests no increased risk of severe disease in immunosuppressed patients [65].

Hepatic involvement in COVID-19 may be part of multiple organ failure in severely affected patients. The liver might also be involved due to ACE2 expression in liver and bile duct cells [98], or by the effects of hepatotoxic drugs. A study summarising the findings of COVID-19 case series reported increased alanine aminotransferase, aspartate aminotransferase, or bilirubin levels in 15–53% of hospitalised patients [98]. More severe illness is associated with a higher incidence of abnormal liver laboratory findings. In a recently published case series a 29% incidence of liver dysfunction in critically ill COVID-19 patients was reported, although it is not mentioned how liver dysfunction was defined [76]. The clinical relevance of abnormal liver laboratory findings is currently unclear, some authors doubt their importance [99]. Hepatotoxicity is a known side effect of lopinavir/ritonavir, may complicate chloroquine and hydroxychloroquine use, and may possibly also occur in remdesivir use. Hepatic dysfunction may require cessation or dosing adjustment of drugs used in COVID-19 [100]. Lopinavir/ritonavir is contraindicated in severe liver dysfunction. In ongoing trials (clinicaltrials.gov) of remdesivir the presence of alanine aminotransferase or aspartate aminotransferase levels above five times the upper level of normal is a contraindication for inclusion.

## 10. Are Ibuprofen or Other Non-Steroidal Anti-Inflammatory Drugs (NSAIDs) Contraindicated in COVID-19 Patients?

The role of NSAIDs and in particular ibuprofen is a good example for a “breaking news” having a higher “reproduction number” than the virus itself. It originates from reports in French media that four young patients with COVID-19 deteriorated after taking ibuprofen, leading to a recommendation to use paracetamol instead of ibuprofen by the minister of health in France [101]. However, the hospital where the information of the four young patients came from officially refused to participate in such speculations.

Furthermore, ibuprofen has respiratory and cardiovascular adverse effects which are relevant to patients at high-risk during a SARS-CoV-2 infection. The Centre for Evidence-Based Medicine of the University of Oxford generally questions the routine anti-pyretic administration of both paracetamol and NSAIDs in acute respiratory infections and COVID-19 [102]. Their doubt is based on results showing that NSAIDs have only a limited symptom-alleviating effect in acute respiratory infections [103] and on retrospective data demonstrating that NSAID use during acute respiratory infections was associated with an increased risk of myocardial infarction and stroke [104,105]. There are presently no data to encourage or discourage the use of ibuprofen and other NSAIDs during a SARS-CoV-2 infection; anaesthetists are well aware of effects and side effects of NSAIDs and are proficient in making well-informed decisions when to use them.

## 11. Conclusions

By giving answers to the above questions that were intensely discussed within our specialism, we hope to help colleagues in clinical decision-making. With ongoing treatment of severely ill COVID-19 patients other questions will come up, e.g., possible cardiac manifestations of the infection and influences of cardio-vascular co-morbidities on the course of the illness, whether plasma from convalescent patients could be used for therapy [106], or whether coagulation disorders are significantly influencing necessity for critical care treatment and mortality [72,79,107]. While respective guidelines on these topics will serve clinicians in clinical practice [108], regularly updating all guidelines concerning COVID-19 will be a necessary, although challenging task in the upcoming weeks and months. For the United Kingdom, the Centre for Evidence-Based Medicine, University of Oxford is summarizing and disseminating the current evidences on COVID-19 on a daily basis. All recommendations during the current extremely rapid development of knowledge must be evaluated on a daily basis, as suggestions made today may be out-dated with the new evidence available tomorrow.

## Figures and Tables

**Table 1:** Comparison of ARDS features in Covid-19 patients and non-Covid-19 patients.

COVID-19 ARDS	Classic ARDS as Response to Systemic Infection or Inflammation
Focal, i.e., non-recruitable lung lesions	Non-focal, recruitable lung lesions
Lower PEEP	Higher PEEP
	Recruitment manoeuvres
Intermediate tidal volumes	Low tidal volumes
Early prone positioning	Prone positioning later during process, as rescue option

ARDS: acute respiratory distress syndrome; PEEP: positive end-expiratory pressure.

**Table 2:** A useful checklist performed during nursing shifts.

Check Hourly, Report with Every Nursing Shift or More Frequent	What and How to Adjust
V_T;_ report absolute V_T_, and V_T_ in ml per kg predicted bodyweight	Is V_T_ sufficiently low, i.e., < 6 mL/kg predicted bodyweight? If the driving pressure is >15 cm H_2_O, or rises, consider further limitation of V_T_;
FiO_2_ and PEEP; report changes over last hours	Are FiO_2_ and PEEP sufficiently low? PEEP higher than 10–12 cm H_2_O is generally not necessary. Reduction of PEEP should be considered if the driving pressure is > 15 cm H_2_O, or rises
Prone positioning; report start, planned time of turning	Is the prone position applied correctly (i.e., sufficiently long); and agree with the nursing staff on the time for turning;
Any deviations; reasons, solutions	If deviated from the above, the rationale should be documented in the record files;
Any patient-specific issues	In individual cases, specific interventions might have shown to be beneficial to the patient, such as a certain positioning (like a left or a right front crawl position when in the prone position), need for use of short–term additional sedation or muscle relaxation (the colleagues in the next shift can use this information and apply the interventions correctly).

V_T_: tidal Volume; FiO_2_: inspiratory oxygen fraction.

**Table 3:** Cohorts including data on diabetes mellitus in the current literature.

Cohort	Hospital	Size of Cohort (*n*)	Percentage Patients with Diabetes Mellitus
Wuhan [71]	Jin Yin-tan	99	12%
Wuhan [72]	Jin Yin-tan	41	20%
Wuhan [73]	Zhongnan	138	10%
Wuhan [74]	Jin Yin-tan + Wuhan pulmonary	191	19%
Wuhan [75]	No. 7	140	12%
Wuhan-ICU [76]	Jin Yin-tan	52	17%
China [77,78]	-	72,314	5%
China [79]	-	1099	7%
Washington State [82]	Evergreen	21	33%
Italy [83]	-	355	36%
Singapore [84]	-	18	6%
Netherlands ^a^	-	2510	13%

a: RIVM. Epidemiologische Situatie COVID-19 in Nederland 25 Maart 2020.
